# Motor cortex latent dynamics encode arm movement direction and urgency independently

**DOI:** 10.1101/2023.05.26.542452

**Published:** 2023-05-26

**Authors:** Andrea Colins Rodriguez, Matthew G. Perich, Lee Miller, Mark D. Humphries

**Affiliations:** 1 School of Psychology, University of Nottingham, Nottingham, United Kingdom; 2 Département de neurosciences, Faculté de médecine, Université de Montréal, Montréal, Canada; 3 Québec Artificial Intelligence Institute (Mila), Québec, Canada; 4 Northwestern University, Department of Biomedical Engineering, Chicago, USA

## Abstract

The fluid movement of an arm is controlled by multiple parameters that can be set independently. Recent studies argue that arm movements are generated by the collective dynamics of neurons in motor cortex. But how these collective dynamics simultaneously encode and control multiple parameters of movement is an open question. Using a task where monkeys made sequential, varied arm movements, we show that the direction and urgency of arm movements are simultaneously encoded in the low-dimensional trajectories of population activity: each movement’s direction by a fixed, looped neural trajectory and its urgency by how quickly that trajectory was traversed. Network models showed this latent coding is potentially advantageous as it allows the direction and urgency of arm movement to be independently controlled. Our results suggest how low-dimensional neural dynamics can define multiple parameters of goal-directed movement simultaneously.

## Introduction

We perform highly skilled arm movements from delicate strokes while drawing to ballistic swings while playing tennis, each of which requires the control of multiple parameters. Motor cortex is thought to play a key role in controlling these parameters (Churchland and Shenoy, 2007; Churchland et al., 2012; Gallego et al., 2017; Russo et al., 2018; Vyas et al., 2020), but it is unclear how they are simultaneously encoded. A promising approach to understanding motor cortex is to study the coordinated activity of populations of neurons (Churchland et al., 2010, 2012; Michaels et al., 2016; Gallego et al., 2017; Glaser et al., 2018; Perich et al., 2018b; Even-Chen et al., 2019; Gallego et al., 2020). Here we address the problem of how a population of neurons in motor cortex can simultaneously encode and potentially control multiple movement parameters.

Motor cortex is increasingly viewed as a dynamical system, whose low-dimensional dynamics generate movement (Churchland et al., 2010, 2012; Shenoy et al., 2013; Michaels et al., 2016; Gallego et al., 2017, 2018; Russo et al., 2018; Perich et al., 2018b; Even-Chen et al., 2019). This view can account for many aspects of motor cortical activity including complex single neuron responses during simple movements (Churchland et al., 2012; Hennequin et al., 2014) and the changes in neuron tuning between preparation and execution (Churchland et al., 2010; Elsayed et al., 2016). It also predicts that much of the neural activity in motor cortex reflects the ongoing evolution of the dynamical system and not the coding of movement parameters (Shenoy et al., 2013; Kaufman et al., 2014; Gallego et al., 2018; Russo et al., 2018). But if the low-dimensional dynamics in motor cortex are to generate movement, then changes in those dynamics should relate to changes in the parameters of movement (Russo et al., 2018; Humphries, 2021; Saxena et al., 2022).

Arm movements are well-suited to testing this prediction as they have parameters that can be independently specified (Gordon et al., 1994; Favilla and De Cecco, 1996; Vindras et al., 2005). An arm movement towards a target has a spatial component, its direction and distance, and an urgency component, its speed or duration. For example, subjects can readily adapt the speed of arm movements to reach a target at a fixed distance and direction (Soechting and Lacquaniti, 1981; Nishikawa et al., 1999; Mazzoni et al., 2007). This independence predicts that if the low-dimensional dynamics of motor cortex generates arm movements, then changes in those dynamics should be independently specifiable between the spatial and urgency components of a reach.

There is considerable evidence that neural activity in motor cortex reflects the direction (Georgopoulos et al., 1982, 1986; Ashe and Georgopoulos, 1994; Moran and Schwartz, 1999; Churchland et al., 2012; Golub et al., 2014; Michaels et al., 2016; Russo et al., 2018) and the urgency (Schwartz, 1992, 1994; Moran and Schwartz, 1999; Paninski et al., 2004; Churchland et al., 2006; Wang et al., 2007; Inoue et al., 2018; Saxena et al., 2022; Schroeder et al., 2022) of arm movement. From a dynamical systems view, researchers have observed that different directions of arm movement are reflected by different movements of the population’s neural activity in a low-dimensional space (Santhanam et al., 2009; Churchland et al., 2012; Michaels et al., 2016; Russo et al., 2018; Even-Chen et al., 2019). And recent work has shown that the differences in the speed of movement alone also correlate with shifts in the low-dimensional dynamics of motor cortex (Saxena et al., 2022). But key elements of the above predictions remain unaddressed: how changes in multiple parameters of arm movement map to changes of the low-dimensional dynamics in motor cortex, and whether those changes in dynamics can be independently specified.

To address these questions, we analysed population activity in motor cortex from monkeys performing a sequential-target task that extensively sampled variations in the direction and urgency of two-dimensional arm movements. We report here that while the direction of movement is encoded by the direction of the trajectory of population activity in a low-dimensional space, the urgency of movement is encoded by how quickly that neural trajectory is traversed. We then show that the direction of a low-dimensional trajectory and the rate of its traversal can be independently controlled by different parameters of a cortical network. In this way, the simultaneous yet independent encoding of the direction and urgency of movement in a population of motor cortex neurons potentially allows them to be independently specified.

## Results

**Figure 1.**
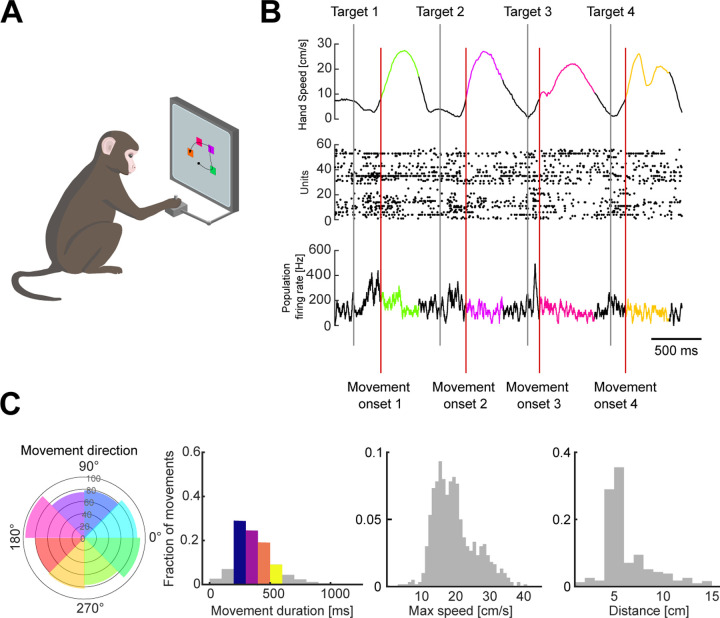
Variability of arm movement behaviour in a sequential-target task. A) Task schematic. Monkeys were trained to reach four targets that appeared sequentially on the screen, using a planar manipulandum (image adapted from (Colins Rodriguez, 2021)). B) Hand speed (top), raster plot (middle), and firing rate (bottom) for an example trial. Grey and red vertical lines indicate the times of target appearance and movement onset, respectively. Highlighted segments show movement execution coloured by movement direction. C) Variability of arm movement parameters for an example session (from left to right): movement direction (radius shows the number of movements), duration, maximum speed and distance. Coloured direction slices show the bins used for direction-based analyses. Coloured bars for duration indicate the bins used for duration-based analyses.

Three monkeys were trained to move a cursor to targets on a screen using a planar manipulandum (Figure 1A). In each trial, four targets appeared sequentially and the monkey had to move the cursor to each in turn before receiving a liquid reward at the end of each trial. Each target’s position was chosen by a pseudo-random algorithm, while its time of appearance was triggered after the monkey reached the previous target and held for 200 ms (Figure 1B). We consider henceforth each movement of the cursor from a starting position to reaching the target as an individual arm movement. Extracellular neural activity from dorsal premotor cortex (PMd) and/or primary motor cortex (M1) was recorded during the task using Utah arrays. We analyse here 6 PMd and 7 M1 population recordings from 10 task sessions, containing between 24 and 95 units.

This task design induced independent variability in the direction, distance, and urgency of arm movements (Figure 1C), allowing us to probe the encoding properties of multiple movement parameters simultaneously. The variables of distance, duration, and maximum speed are naturally correlated during arm movements (Figure 1–Figure Supplement 1), yet their degree of correlation varied in this task: for instance, movement duration depends on speed and distance but had a stronger correlation with distance (mean *R* = 0.56) than with maximum speed (mean *R* = 0.27, Figure 1–Figure Supplement 1). Distance and maximum speed were strongly correlated (mean *R* = 0.8, Figure 1–Figure Supplement 1). As our goal was to address how motor cortex could independently represent the spatial and urgency components of arm movement, we thus focused on the most independent of those parameters in the task, respectively movement direction and movement duration.

### Arm movement direction is encoded in the trajectory of population activity

To analyse how arm movement direction and duration were simultaneously encoded in the population, we binned the movements into eight directions and four durations (a total of 32 conditions), as shown in Figure 1C. Neural activity from each movement was aligned to movement onset, defined as the time at which the hand’s speed crossed a threshold of 8 cm/s. For each movement, we selected the corresponding neural activity from 500 ms before movement onset until 300 ms after the movement ended.

**Figure 2.**
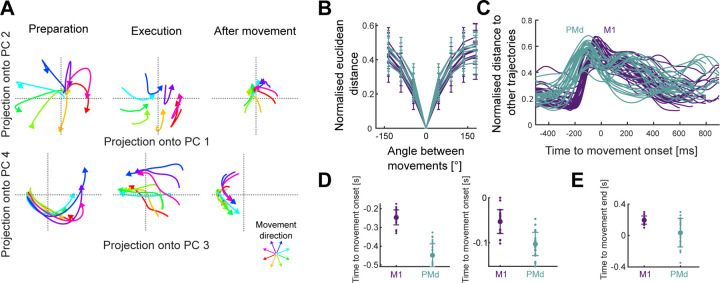
Neural trajectories encode arm movement direction. A) Direction-dependent neural trajectories of movements between 200 and 300 ms duration from an example M1 recording. Neural activity was included from 250 ms before movement onset until 200 ms after movement end and divided into 3 stages: preparation, up to movement onset (left); execution, between movement onset and movement end (middle); and after movement, from when the hand reached the target (right). B) Mean distance (across time) between neural trajectories as a function of the angle between the corresponding arm movements (of 200–300 ms duration). One line per recording. Error bars indicate SD. Distances were normalised by the maximum distance between two points in each recording. C) Mean distance over time (across directions) between trajectories for the same movement duration (N=52 lines: 4 duration bins × 13 recordings). D) Times of minimum (left) and maximum (right) distance between trajectories during preparation. E) Time of minimum distance between trajectories after movement onset.

For each recording, we found the average neural activity for each of the 32 conditions, then performed PCA across all conditions to define a common subspace. We defined the embedding dimensions of the subspace as the number of dimensions required to explain at least 80% of the variance of the neural activity in that recording (range: 3–11 dimensions across recordings; Figure 2–Figure Supplement 1). Projecting a recording’s population activity into the defined subspace showed how it evolved over time for each direction of movement (Figure 2A), which we call here its neural trajectory.

Across all recordings, we observed two common features: Neural trajectories separated from each other according to the direction of movement, and they all returned to the same region of the subspace after the end of movement (Figure 2A). To quantify their separation, we measured the average Euclidean distance between trajectories corresponding to different directions and the same duration. The distance between trajectories in both M1 and PMd was proportional to the angle between movements (Figure 2B), consistent with the direction of arm movement being encoded by the trajectory of neural activity.

We then asked how the distance between neural trajectories corresponding to different directions (and the same duration) evolved over time. There was a marked difference in the timing of the trajectories in M1 and PMd activity (Figure 2C). During preparation, trajectories in PMd reached a minimum separation between each other ~200 ms before the trajectories in M1 did (Figure 2D, left). The maximum separation of trajectories also happened first in PMd (Figure 2D, right). After movement onset, PMd trajectories also returned to their minimum separation before M1 trajectories did (Figure 2E). Together, these results suggest that a series of movement-related changes to neural activity in PMd consistently precede the corresponding changes in M1. To directly compare the trajectories in PMd and M1, we henceforth considered the neural trajectories exclusively between the average times of their minimum separation before movement onset and after movement end (450 ms before movement onset to 50 ms after the end for PMd; 250 ms before movement onset to 200 ms after the end for M1).

**Figure 3.**
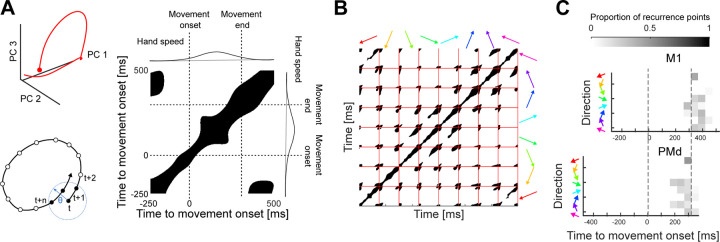
Trajectories of motor cortical activity start and end in the same region of subspace. A) Recurrence analysis. Consider an example trajectory of M1 activity (top left) from 250 ms before movement onset (circle) to 500 ms after onset. In this three-dimensional projection, the start and end of the trajectory seem to occur in the same region of subspace. To test this in the full *n*-dimensional subspace, we define that a point **p**(*t*) on the trajectory *recurs* (schematic, bottom left) if a later point **p**(*t* + *n*) is within some small distance *θ* from **p**(*t*). If it is, the time *n* is the recurrence period of the trajectory. Testing all points **p**(*t*) from 250 ms before movement onset to 500 ms after onset gives us a recurrence plot (right): a matrix in which black squares indicate pairs of times when the trajectory recurred. B) All recurrence plots for an M1 recording, for movements of 200–300 ms duration. Plots on the diagonal are for the neural trajectory for each direction of movement (arrows) – plot in (A) is the bottom-left corner. Off-diagonal plots are the cross-recurrence between the trajectories for two different directions: black regions here show times when the trajectory for one direction passes within *θ* of the other. Note the black regions in the bottom right and/or top left of every plot, showing that trajectories for two different directions recurred at or after the end of movement. C) Histograms of the periods between a trajectory’s start and its recurrence, for one example recording. Trajectories are for movements of 200–300 ms duration.

While the preceding analysis showed that neural trajectories for different directions of arm movement start and end close together (Figure 2C–E), it could not tell us if their common end point was in the same region of the subspace as their starting point. To test this, we used recurrence analysis (Figure 3A; see Methods). Figure 3A, right panel, shows an example recurrence plot for a trajectory in M1 associated with one direction and duration of arm movement, with the black regions indicating the times where the trajectory recurred: the black regions at around 450 ms after movement onset show that the trajectory of activity in M1 returns to the same region of subspace it was in around 200 ms before the movement started.

This recurrence of neural trajectories to the same region of subspace before and after movement occurred for each direction of arm movement (diagonal of Figure 3B). Moreover, the starting and ending regions within the subspace were the same for trajectories corresponding to every pair of different directions (off-diagonal of Figure 3B). The period of this recurrence was consistent across movement directions of the same duration, and PMd activity recurred earlier than M1 activity as expected (Figure 3C; Figure 3–Figure Supplement 1 shows histograms for all recordings). We further confirmed that movements of different duration in the same direction had neural trajectories that started and ended in the same region of activity subspace (Figure 3–Figure Supplement 2). Collectively, these data support the hypothesis that the direction of arm movement is encoded in the direction the trajectory of neural activity takes as it moves away from and returns to a common region of neural subspace.

**Figure 4.**
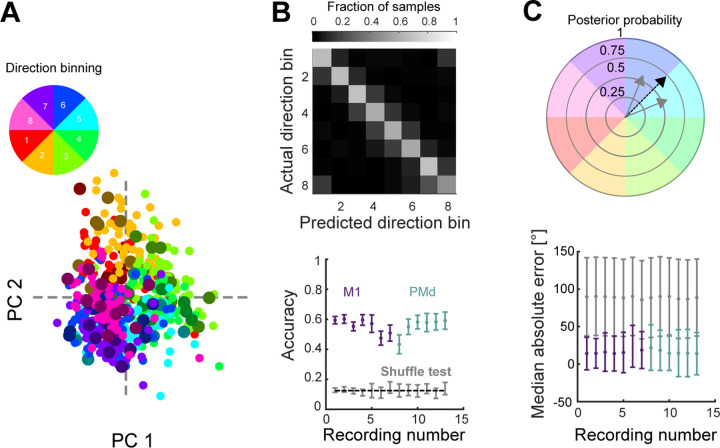
Movement direction can be well decoded from neural trajectories before movement onset. A) Projection of average preparatory neural activity (200 ms window before movement onset) onto the first 2 PCs from one recording. Each dot is the projection of the average neural activity during movement preparation. Small, bright dots show training data; big, darker dots show test data. B) Classifier performance. Top: Confusion matrix for one example recording, each element showing the agreement between prediction and data. Bottom: cross-validated accuracy of the classifier for all recordings. Dashed line shows chance (1/8). Bars indicate SD. C) Continuous estimation of movement direction. Top: schematic of angle estimation. Given a neural trajectory, we found the posterior probability that the trajectory belonged to each direction bin (grey solid arrows show two non-zero probabilities in this example). We then estimated the movement direction encoded by the trajectory as the sum of the vectors corresponding to each bin (black dashed arrow). Bottom: Median error of the predicted direction for all recordings. Grey dots and bars show the direction shuffled data.

To further test this hypothesis, we asked if we could decode movement direction during movement preparation, when the trajectory of the population first departs the common region of subspace. We trained a Naive Bayes classifier to predict the direction of the upcoming movement (from the eight binned directions) from a PCA projection of the average neural activity (over time) in the 200 ms window before movement onset (Figure 4A). The classifier reliably predicted the held-out direction bin for all monkeys and recordings (Figure 4B, average accuracy M1=0.55; average accuracy PMd=0.56; chance = 0.125).

We next asked how well the specific angle (within the direction bin) of the upcoming movement could be predicted. For this, we represented the output of the classifier as 8 vectors, corresponding to the mean angle of each direction bin. The length of these vectors was given by the posterior probability of each direction bin found by the Naive Bayes classifier. The predicted angle was then defined as the angle of the sum of all vectors (Figure 4C, top). This method could accurately predict the upcoming movement direction in all recordings, improving the median absolute error of the decoder from 20° (using the mean angle of the predicted bin) to 16° (Figure 4C, bottom). Direction of arm movement could thus be well decoded from the trajectory of neural activity as it moved away from a common region of the subspace.

### Arm movement urgency is encoded in the timing of the neural trajectories

**Figure 5.**
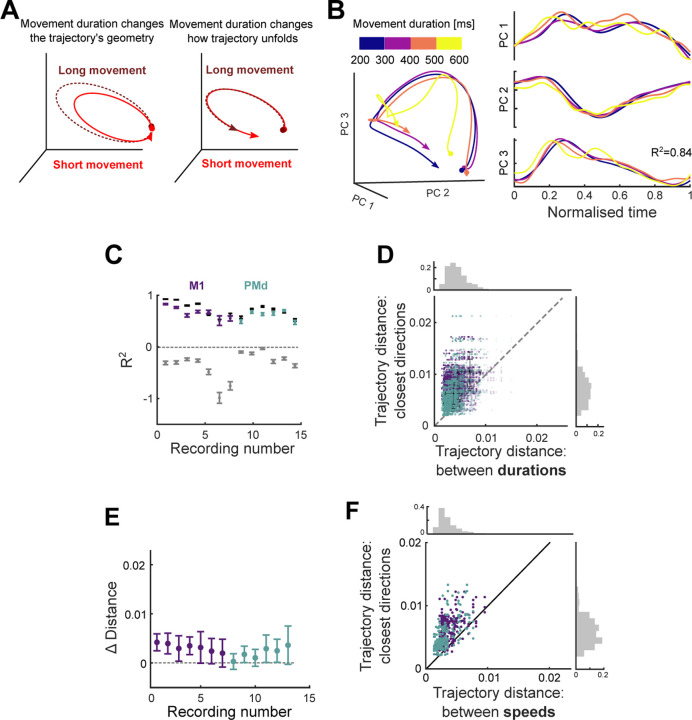
Movement urgency is encoded by the traversal of fixed neural trajectories. A) Hypotheses for changes in neural trajectories due to variations in movement urgency, illustrated for duration: movement duration changes the geometry of neural trajectories (left) or how the trajectory unfolds over time (right). Hypotheses were selected to be consistent with trajectories that recur to the same region of the subspace (Figure 3). B) Left: Projection of neural trajectories corresponding to the same direction but different movement duration, all plotted for 600 ms. Right: Trajectories from the left panel normalised by their duration. C) Coefficient of determination *R*^2^ of time-scaled trajectories corresponding to the same direction (coloured). Grey bars give the estimated lower limits for *R*^2^ from time-scaled trajectories corresponding to all different directions for each recording. Black bars give the estimated upper limit for *R*^2^ from sub-sampled trajectories from the same duration and direction bin. Error bars indicate the standard error of the mean (SEM). D) Hausdorff distance between trajectories from adjacent direction bins compared to Hausdorff distance between trajectories corresponding to different durations of movements in the same direction. Opacity of each data point is proportional to the number of samples used to produce each trajectory. Solid colour shows maximum number of samples. Black error bars indicate mean and SD for each recording. Top histogram shows the distribution of the distance between trajectories corresponding to different durations. Right histogram shows the distribution of the distance between trajectories corresponding to adjacent direction bins. E) Difference between the distance between trajectories corresponding to adjacent direction bins (*y* axis in panel D) and the distance between trajectories with different durations (*x* axis in panel D). Error bars indicate mean and SD for each recording. F) Same as D), for trajectories corresponding to different speeds.

We then asked if and how movement urgency, quantified here by movement duration, could be simultaneously represented by the same population of neurons. We considered two hypotheses. First, the *geometry* hypothesis: for a given direction, movement duration could be represented by changes in the path of the neural trajectory, for instance, longer movements could be generated by larger trajectories (Figure 5A, left). Alternatively, the *scaling* hypothesis, that the neural trajectory for a given direction of movement is fixed, and movement duration is represented by the speed at which that fixed trajectory is traversed (Figure 5A, right): the slower the neural trajectory, the longer the movement.

We found that differences in movement duration affected the geometry of the neural trajectories only minimally, but were instead reflected in the scaling of a fixed trajectory. Figure 5B (left) shows that neural trajectories corresponding to the same direction yet different durations of arm movement closely overlap each other when they are all plotted over the same time period. When trajectories are normalised by their duration, so movements of different durations can be compared, their shape is strikingly similar (Figure 5B, right). To quantify this, we computed the coefficient of determination (*R*^2^) between the time-scaled trajectories corresponding to the same direction and different durations (Figure 5C). For all recordings, we found that the average *R*^2^ for scaled trajectories corresponding to the same direction (mean *R*^2^ = 0.64) was significantly higher than the average *R*^2^ between scaled trajectories corresponding to different directions (mean *R*^2^ = −0.33; *t*-test for each recording, max p-value<10^−6^, *N*(same direction) = 48, *N*(different directions) = 336). Trajectories for the same direction but different durations of arm movement were thus considerably more similar to each other than were trajectories for different directions.

To see how well scaling the trajectories could account for all their variation across different durations, we also estimated an upper limit for *R*^2^ by computing it between neural trajectories from movements of the same duration and direction bin (black bars in Figure 5C; Methods). The average upper limit we obtained (*R*^2^ = 0.72 over all recordings) was close to that for the scaled trajectories corresponding to different durations (*R*^2^ = 0.64). Indeed, for 7 of the 13 recordings we found no significant difference between the estimated upper limit of *R*^2^ and the *R*^2^ for scaled trajectories (*t*-test for each recording, non-significant p-values 0.16 − 0.7, M1=3, PMd=4. Significant p-values 10^−6^ − 0.04, M1=4, PMd=2). Across all recordings the scaled trajectories accounted for at least 92% of the difference between our estimated lower and upper limits for *R*^2^ (Figure 5C). Consequently, nearly all the variation in neural trajectories between different durations of arm movement could be accounted for by the rescaling of a fixed trajectory.

The scaling hypothesis further predicts that differences in the duration of movement would have a smaller impact on neural trajectories than differences in direction. To test this, we compared the distances between neural trajectories for movements of differing duration to those of differing (adjacent) directions. For all recordings, we found the distance between trajectories corresponding to different durations to be significantly smaller than the distances between trajectories from adjacent direction bins (Figure 5D–E, *t*-test for each recording, max p-value=3 · 10^−3^, *N* = 192), indicating that changes in movement duration had only a small effect on the geometry of neural trajectories in M1 and PMd. Collectively, these results support the scaling hypothesis, that duration is encoded by the rate at which population activity follows a fixed trajectory (Figure 5A, right).

If different durations are encoded by the rate a neural trajectory is traversed, it follows that different speeds of arm movement should be too. Because arm movement distance and speed were strongly correlated (Figure 1–Figure Supplement 1), we selected a subset of movements for which distance was within a narrow range (see Methods). We then binned the neural activity into high and low speeds (averages 14 [cm/s] and 19 [cm/s] respectively) and the same 8 direction bins. We projected the neural activity into the common subspace defined above (2 speeds × 8 directions = 16 conditions total) and compared the distances as in Figure 5D. For all recordings, the distance between trajectories corresponding to different speeds was significantly smaller than the distances between trajectories from adjacent direction bins (Figure 5F. *t*-test for each recording, max p-value=3 · 10^−6^, *N* = 32). This suggests that, for movements whose distance is fixed, the speed of the movement induces minimal changes in the geometry of the trajectories, consistent with the encoding of movement urgency in how quickly a fixed trajectory of neural activity was traversed.

**Figure 6.**
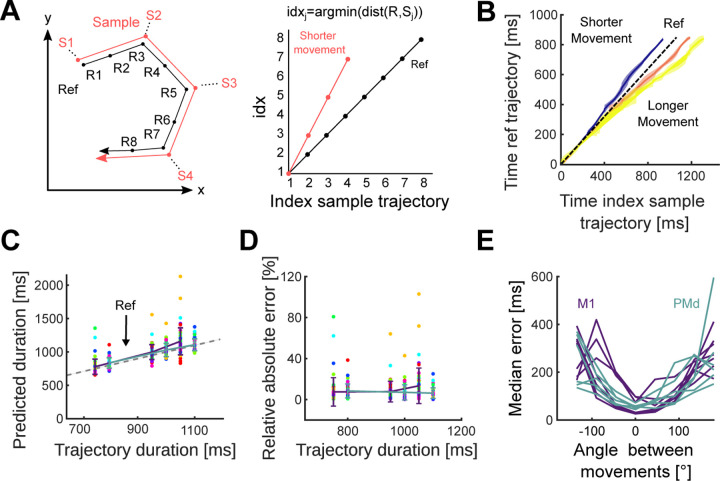
Movement duration can be decoded from the traversal of a fixed trajectory. A) Schematic of the decoder for duration. Left: a sample trajectory was compared to a reference trajectory of the same direction. Right: time indices at which each point on the sample trajectory is closest to the reference trajectory. We estimated the relative speed (*v*) of the sample trajectory compared to the reference trajectory as the slope of a linear fit to the time index plot, and decode *Duration*_*sample*_ = *Duration*_*ref*_ *v*. B) Time indices for sample trajectories from an example recording. Using the 300–400 ms duration movements as a reference (black), time indices were calculated for movements in the other three duration bins. Each coloured line and area show the mean time index and standard deviation (across directions) for each movement duration. C) Predicted versus actual trajectory duration for all recordings. Coloured error bars indicate mean and SD for M1 and PMd (key in panel E). Coloured circles indicate different movement directions. The dashed line indicates the actual value of trajectory duration. D) Relative error of decoder predictions in panel C. E) Decoder error when the reference and sample trajectories are from movements in different directions. The x-axis plots the angle between the movements of the reference and sample trajectories. Each line shows a recording.

To further test the scaling hypothesis, we designed a decoder that explicitly assumed duration is encoded by the rate at which neural activity followed a fixed trajectory. We estimated the duration of a sample trajectory (red in Figure 6A, left) by comparing it to a reference trajectory (black in Figure 6A, left) for the same movement direction. If the sample and reference trajectories were indeed the same, then the time at which the sample trajectory reached a particular point in the subspace should consistently lead (if faster) or lag (if slower) the time at which the reference trajectory reached the same point. We should thus be able to decode the duration of the sample trajectory simply by finding its relative speed compared to the reference trajectory (Figure 6A, right; Methods). For each recording, we defined its reference trajectory as the trajectory associated with movements that lasted between 300 and 400 ms. We observed a consistent delay between the sample trajectories and this reference trajectory (Figure 6B). Consequently, our linear decoder accurately predicted trajectories’ duration across all recordings (average relative error: 9.5% in M1 and 7.2% in PMd, Figure 6C,D).

To further test the separability of the encoding of movement direction and duration, we tested duration decoding when using a reference trajectory of a different direction of movement. The error of the duration predictions increased with the angle between the directions corresponding to the reference and sample trajectories (Figure 6E), providing further evidence that duration is encoded by the rate at which population activity traverses a fixed trajectory.

### Direction and urgency can be independently controlled

We then examined how the independent encoding of direction and urgency could be implemented by a neural population, and how this would allow control over those parameters. For this, we built a recurrent neural network based on a stability-optimised circuit model (Hennequin et al., 2014) (Methods). This type of network is composed of *n* excitatory and inhibitory neurons whose synaptic connection strengths are optimised to produce a balanced, non-chaotic network. These networks exhibit strong transient activity and are able to reproduce fundamental features of motor cortex dynamics at single-cell and population levels, such as the multi-phasic firing rate responses of individual units and the rotational trajectories in their low-dimensional subspace (Churchland et al., 2012; Hennequin et al., 2014). This type of network is not trained to produce outputs that resemble the neural activity; instead, the constraints imposed on the dynamics of the network allow the reproduction of key features of the neural activity.

**Figure 7.**
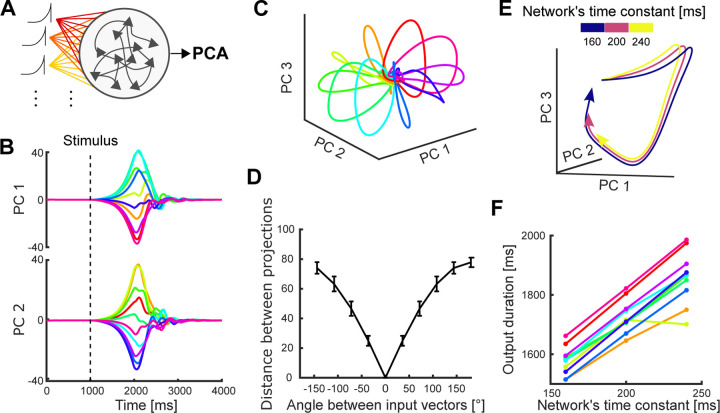
Independent control of movement direction and duration in a recurrent neural network (RNN). A) Schematic of an RNN whose input is rotated to simulate different movement directions. Population activity of the neural network is used to define a low-dimensional subspace analogous to those defined from the PMd and M1 unit activity. B) Temporal projection of network activity. Each colour indicates a rotation of the input. Dashed line shows the time of the stimulus’ start. C) Neural trajectories of an example network for each of the ten rotations of the input, simulating ten different target directions of arm movement. D) Average Euclidean distance between the network’s neural trajectories corresponding to different input rotations. Error bars show standard deviation. E) First 1.9 s of the RNN’s neural trajectories for a single input rotation while varying the network’s time constant. F) Estimated duration of the neural trajectories for different time constants, one line per input rotation. Duration was estimated by the recurrence of the trajectory to the starting region of the subspace.

Following Hennequin et al. (2014), we modelled the input to the network as a ramp and rapid decay, emulating the preparatory period. The input was projected into the network by an n-dimensional weight vector randomly chosen from a uniform distribution ([−1, 1]). To simulate the generation of trajectories corresponding to different arm movement directions, we rotated the input projection vector to span 360° in 10 directions (Figure 7A, see Methods). We then examined the population dynamics of the network by applying the same PCA analysis as the data across the responses to all 10 directions.

We found clear rotational and recurrent dynamics in all trajectories, similar to the dynamics we observed in M1 and PMd (Figure 7B and Figure 7C). We also observed clean separation between the trajectories (Figure 7C). Furthermore, the distance between the trajectories generated by the network was proportional to the angle between input vectors (Figure 7D), analogous to how the distance between neural trajectories from M1 and PMd was proportional to the difference in the angle of arm movement (Figure 2B). We found similar results for inputs chosen in the range [0, 1] (Figure 7–Figure Supplement 1), modelling solely excitatory inputs to the cortical circuit. Therefore, the separation of neural trajectories observed in motor cortex can be induced by rotating the projection of a common input signal.

We then examined how the same network could independently control the rate at which the neural trajectories were traversed. For this, we generated trajectories using a fixed input projection while varying the time constant of the network (Eq.2, Methods), which determines how quickly its neurons respond to their inputs. We found that trajectories generated with larger time constants were slower with preserved shape (Figure 7E), analogous to the neural trajectories corresponding to different movement durations we observed in motor cortex (Figure 5B). We confirmed that this slower rate translated into a longer duration by defining a region of recurrence around the steady-state of all trajectories, similar to the recurrence region defined for neural trajectories (Figure 3–Figure Supplement 2), and comparing their time of recurrence (Figure 7F). Therefore, changing the timing of trajectories while preserving their shape can be induced by changing the time constant of the network.

## Discussion

The dynamical systems view of motor cortex proposes that its low-dimensional dynamics generate movement (Churchland et al., 2010, 2012; Shenoy et al., 2013; Michaels et al., 2016; Gallego et al., 2017; Russo et al., 2018). This view leaves two open questions: of how changes in the system’s dynamics map to changes in the parameters of movement (Russo et al., 2018; Saxena et al., 2022); and of how those dynamics might be controlled to cause the change of their corresponding parameters of movement.

To address the first question, we took advantage of the variable direction and duration of arm movements naturally occurring in a sequential target task. This allowed us to address whether parameters that can be specified independently in behaviour are also encoded independently in the dynamics of motor cortex. We found that they were: the direction and duration of arm movements respectively mapped to the direction and rate of traversal of low-dimensional trajectories of motor cortex activity.

Previous work reported that qualitatively different two-dimensional arm movements corresponded to different trajectories of low-dimensional neural activity (Santhanam et al., 2009; Churchland et al., 2012; Even-Chen et al., 2019). We extended this in two ways. First, we established a quantitative relationship between neural activity and movement direction, by showing that the distance between neural trajectories is proportional to the angle between the corresponding arm movements (Figure 2). Second, we showed that such direction encoding is transient as trajectories start and end in the same low-dimensional region irrespective of the direction they encode. As a trajectory in space must rotate to end up back where it started, this may explain why population activity in motor cortex shows rotations during the onset of straight-line arm movements (Churchland et al., 2012).

Temporal scaling of neural activity has been previously observed in prefrontal cortex (Wang et al., 2018; Remington et al., 2018) and in the striatum (Gouvêa et al., 2015) when animals are required to judge the duration of time intervals. However, in the same setting, temporal scaling is not present in the thalamus (Wang et al., 2018), and so is not an inevitable property of neural dynamics in a time-judgement task. Conversely, as the sequential-target task analysed here presented minimal time constraints, our results also show that temporal scaling of neural activity can be observed in the cortex even when there is no explicit timing required by a task.

Our observation of these fixed neural trajectories that preserved their shape across variations in movement speed (Figure 5F) may seem at odds with a recent report of arm movement speed altering the trajectories of neural activity (Saxena et al., 2022). This difference could be explained by the selection of the parameters examined in each analysis: Saxena et al. (2022) analysed the effects of the speed of a cyclical arm movement separately for each direction of movement, so isolating the effects of speed on the trajectory of neural activity. We examined both direction and urgency (including speed) together and found that direction had a much larger effect on the neural trajectory: so while we cannot say that the urgency of movement had no effect on the trajectory of neural activity, our results suggest that the difference in the trajectory of neural activity between any two movements is dominated by differences in their direction rather than their urgency. Alternatively, the point-to-point arm movements of the target task analysed here and the repeated rotational movements of the cycling task used by Saxena et al. (2022) could be encoded differently within motor cortex, perhaps within different subspaces (DePasquale et al., 2023), such that the speed of rotation in the cycling task does indeed notably alter the trajectory of neural activity.

We found that both M1 and PMd encode movement direction and urgency similarly, yet have a clear difference in their timing. Neural activity in PMd preceded activity in M1 by 200 ms on average, in agreement with previous studies (Thura and Cisek, 2014). While our results suggest that PMd and M1 share a coding strategy for movement direction and urgency, they could still each contribute to different stages of movement control, such as preparation and execution. The sequential changes in trajectories between PMd and M1 are consistent with the hypothesis that premotor cortex activity moves M1 activity to the necessary initial state for its autonomous activity to generate arm movements (Churchland et al., 2010; Shenoy et al., 2013; Kaufman et al., 2014); however, as PMd dynamics remained distinct between movements in different directions up until their end point, our results are also consistent with PMd input to M1 playing a role in shaping M1’s ongoing pattern generation during an arm movement (Sauerbrei et al., 2020).

To address the second open question of how low-dimensional dynamics in motor cortex might be controlled to alter the corresponding parameters of movement, we showed that the direction and rate of neural trajectories can be independently controlled in a recurrent neural network model. In our simulations, direction and rate were changed by respectively rotating the vector of inputs and scaling the network’s time constant. These two mechanisms could be implemented in various ways in motor cortex. A recent study showed that the dynamics of motor cortex during planning and execution of movements is highly modulated by external inputs (Sauerbrei et al., 2020). This suggests a rotating input could be implemented by projections from either other cortical areas or the thalamus (Inagaki et al., 2022; Moll et al., 2023). The scaling of neural trajectories, characterised here as a change in the time constant of the network, could also be approximated by modulating the neuronal gain across the population (Stroud et al., 2018). Global gain modulation could occur via neuromodulators (Thurley et al., 2008; Wei et al., 2014); gain modulation could also occur by changing tonic inputs to the network that drive the neurons to their saturating nonlinearity (Remington et al., 2018; Wang et al., 2018). Other network-level mechanisms for controlling the direction and rate of neural trajectories may exist: our goal here was to show proof of principle that independent network-level mechanisms exist to control both observed features in the data.

From our data and modelling results arise three hypotheses for how motor cortex activity can be manipulated to control multiple arm movement parameters simultaneously and independently. First, that the direction of the low-dimensional trajectory of neural activity controls the planar direction of arm movement. Second, that the rate of traversing a specific low-dimensional trajectory of neural activity controls the urgency of an arm movement in that direction. We used duration here as our measure of urgency because of the behavioural statistics of the task we analysed (Figure 1), but consider it unlikely that the duration of an arm movement is explicitly specified. Third, that this separation of encoding allows direction and urgency to be independently specified, as we see in behavioural tasks (Soechting and Lacquaniti, 1981; Nishikawa et al., 1999; Mazzoni et al., 2007), by network-level changes that can act on the time-scale of individual movements.

Further exploration of these ideas could usefully look at the relationship between low-dimensional dynamics and the other crucial parameter of arm movement to a target, distance. One possibility is that motor cortex encodes the target’s position in coordinates centered on the current hand position, from which both direction and distance could be computed. However, behavioural data have long suggested the hypothesis that the direction and extent of arm movements are also independently specified (Ghez et al., 1991; Gordon et al., 1992; Ghez and Krakauer, 2000). This hypothesis arose from behavioural studies showing that the distributions of target errors in arm movements were independent between the direction and extent of movement (Favilla et al., 1990; Gordon et al., 1994; Favilla and De Cecco, 1996; Vindras et al., 2005), and that adapting direction and extent to new targets occurred at independent rates (Krakauer et al., 2000). A fuller model of the mapping between low-dimensional dynamics of motor cortex and changes in movement parameters will thus require determining if and how distance is so encoded.

Nonetheless, our findings here support the hypothesis that low-dimensional dynamics of a population of neurons generate arm movements, and suggest that studying the mapping between low-dimensional dynamics and multiple parameters of arm movement may be as insightful in our understanding of motor cortex as it has been in other motor systems (Bruno et al., 2017; Lindén et al., 2022).

## Methods and Materials

### Code availability

All code used to produce the figures in this paper was developed in MATLAB and is available in our GitHub repository https://github.com/Humphries-Lab/Motor_Cortex_Simultaneous_Coding.

### Data availability statement

All data used to produce the figures in this paper (electrophysiological recordings and simultaneous behaviour) will be available at Dryad upon publication. A subset of this dataset (4 recordings) was previously published at crcns.org at http://dx.doi.org/10.6080/K0FT8J72.

### Subjects and task

We analysed 13 recordings from the motor cortex (6 from PMd and 7 from M1) of three monkeys (Monkey M, T and C). Monkey C contributed 3 recordings from M1, Monkey T contributed 3 recordings from PMd and Monkey M, which had implants in M1 and PMd simultaneously, contributed 6 recordings. This gave a total of 10 behavioural sessions. Data from 4 recordings (monkey M and T, PMd and M1 areas) are publicly available (Perich et al., 2018a).

Monkeys were trained to reach four targets that sequentially appeared on the screen, using a planar manipulandum. Hand movements were constrained to the horizontal plane on a workspace of 20 x 20 cm. Each target’s position was chosen by a pseudo-random algorithm which selected the distance (5–15 cm) and angle (0–360°) with respect to the previous target.

The time of target appearance was triggered after the monkey reached the previous target and held for 100 ms. The targets appeared on the screen on average 96 ms after they were triggered, therefore approximately 200 ms after the monkey reached the target acceptance window. The target acceptance window was a 2 cm square centred on the target position.

We define movement direction for the first reach of the sequence as the direction between the hand position at the moment of target appearance and the first target’s position. For later reaches, the movement direction corresponds to the direction between the positions of the current and next targets.

Only successful trials were used for the following analyses to ensure consistency across reaches between targets. Failed trials by, for example, leaving the centre of the workspace or not holding the target for the required minimum time, were discarded.

All surgical and experimental procedures were consistent with the guide for the care and use of laboratory animals and approved by the institutional animal care and use committee of Northwestern University.

### Neural recordings

Subjects were implanted with 100-electrode arrays (Blackrock Microsystems, Salt Lake City, UT) in PMd (1 mm electrode shaft length) and M1 (1.5mm length). Spike sorting was performed manually using standard techniques. See Lawlor et al. (2018); Perich et al. (2018a); Glaser et al. (2018) for a detailed description of the neural recordings. Each recording contains between 24–95 units. The numbers to identify the recordings are consistent across all figures.

### Data processing

Spike trains were filtered with a Gaussian filter of standard deviation *σ*. For each recording, we defined *σ* as the median of its inter-spike interval (*σ* = 36 − 46 ms for all recordings). We aligned the neural activity to movement onset defined by a hand-movement speed threshold of 8 cm/s (Lawlor et al., 2018). The movement’s end corresponded to the time when the hand entered the acceptance window. The movement duration was defined as the time between the movement’s onset and end.

Arm movements were binned according to their direction (8 bins, as in Figure 1) and duration (4 bins, 200–300, 300–400, 400–500 and 500–600 ms), resulting in 32 conditions. To make all neural activity in a duration bin have equal length, we rounded the duration of the movement to the largest end of their duration bin. For example, if the associated movement lasted 240 ms, then the movement’s end was considered to be 300 ms after movement onset. For each condition, we selected the neural activity from 500 ms before movement until 300 ms after the movement ended (600–900 ms from movement onset) and averaged the neural activity across movements.

### Finding neural trajectories

We performed Principal Component Analysis (PCA) across all conditions’ averaged neural activity to define a common subspace for that recording. Neurons that showed less than 5 spikes across all conditions were discarded. Because the variance of each neuron varies with its firing rate, we balanced the variance across the population by soft-normalising the responses of each neuron (normalisation factor= firing rate range + 5) before applying PCA (Russo et al., 2020; Saxena et al., 2022). The embedding dimensions of each subspace were chosen as the number of Principal Components (PCs) necessary to explain 80% of the variance of the neural activity. For all subsequent analyses, the number of dimensions used is equivalent to the embedding dimensions (Figure 2–Figure Supplement 1).

We obtained the neural trajectories by projecting the population neural activity onto the common subspace. We then calculated the mean distance across all trajectories corresponding to different directions for each movement duration bin. To define the beginning of the movement preparation, we found the time at which the mean distance between trajectories reached a minimum before movement onset (Figure 2D). To define the end of movement encoding, we found the time at which the mean distance between trajectories reached a minimum after movement onset (Figure 2E). For all subsequent analyses, the start and end of the trajectories correspond to the beginning of preparation and end of encoding as defined here.

### Recurrence

To test if each trajectory returned to the same region where it started, we used the idea of recurrence (Marwan et al., 2007; Bruno et al., 2017). Considering a point ***p***(*t*) of a trajectory at time *t*, where ***p*** is a vector of dimension equal to the embedding dimensions, it is said that ***p***(*t*) recurs if another point in the trajectory ***p***(*t* + *n*) passes within some small threshold distance *θ*. If it does then *n* denotes the recurrence time of *t*. When checked over all possible pairs of times *t* and *t* + *n*, this process defines a recurrence matrix, where each element indexes a pair of times and is either a 1 if recurrence occurred between that pair or a 0 otherwise. We calculated the recurrence matrix for all trajectories (concatenated) for movements between 200–300 ms duration. We defined *θ* as the 10^*th*^ percentile of all distances between points in each recording. (Figure 3B).

To calculate the time of recurrence for each trajectory, we counted the number of recurrent points of the first 200 ms of each trajectory in disjoint 50 ms bins (Figure 3C and Figure 3–Figure Supplement 1).

To test that the region of recurrence was invariant to the duration of the movement, we defined a region of recurrence as follows: a *D* dimensional hypersphere centred on the average starting position of all trajectories corresponding to the movements between 200–300 ms. Its radius was equal to the threshold *θ*; *D* is the number of embedding dimensions of the subspace. We then counted how many trajectories from each duration bin were inside the recurrence region at each moment in time (maximum of 8 for each duration bin, Figure 3–Figure Supplement 2)

### Decoding direction

To predict the direction of the upcoming movement from the neural activity, we trained Naive Bayes classifiers based on the projection of the average pre-movement activity of each movement (average in the 200 ms window before movement onset). For this, for each recording, we defined a subspace by applying PCA to all average neural activities and selecting the embedding dimensions as the number of dimensions necessary to explain at least 80% of the variance. We then divided the neural activity samples into 8 classes according to their movement direction. The Naive Bayes classifiers, trained using Matlab (fitcnb), provided the predicted posterior probability for each direction bin given a sample. The predicted class of each sample was the class that maximised the posterior probability. Classifiers’ performance was assessed using 6-fold cross-validation (10 repetitions). Results in Figure 4B and Figure 4C correspond, respectively, to the accuracy and median absolute error pooling folds and repetitions. For comparison, we performed a shuffle test in which the direction bin labels were shuffled, and classifiers were trained and tested on shuffled data.

We also predicted the angle of movement for each upcoming movement by representing the output of the classifier as 8 vectors, whose directions corresponded to the mean angle of each bin, while their length was given by the posterior probability of each bin. Then the predicted angle was defined as the vector resulting from the sum of all vectors. For comparison, we performed a shuffle test in which the angle labels were shuffled.

### Comparing time-scaled trajectories

We temporally scaled the neural trajectories by linear interpolation of their values. All scaled trajectories had 600 time bins. To quantify how well two temporally scaled trajectories matched (Figure 5C), we computed their coefficient of determination (*R*^2^). For this, we collapsed the temporally scaled low-dimensional trajectories ***X*** (composed of points ***x*** of *D* dimensions) and ***Y*** (composed of points ***y*** of *D* dimensions) each into a single vector containing all dimensions concatenated (length ***x*** · *D*). We then computed the coefficient of determination

$$R^{2}\left(X,Y\right)=1-\frac{\sum {\left(X-Y\right)}^{2}}{\sum {\left(X-\bar{X}\right)}^{2}}$$

Where $\bar{X}$ denotes the mean of *X*. Therefore, *R*^2^=1 indicates perfect matching of the trajectories, *R*^2^=0 indicates trajectories match as well as the mean value of the trajectories (baseline) and *R*^2^<0 indicates that matching of the trajectories is lower than the baseline model.

To compare how similar two trajectories corresponding to the same duration and direction bin could be, we randomly divided all the movements corresponding to the same direction and duration bin into two equal-sized sets. We then created two trajectories by taking the average neural activity of each set and projecting them into the low-dimensional subspace. The coefficient of determination was calculated between these two trajectories. We repeated these three steps for half the number of movements corresponding to each duration and direction bin. We then pooled all the values across directions and durations to compute the average of each recording (Figure 5C).

### Comparing distances between trajectories

To compare the distance between trajectories of different lengths, we used the Hausdorff distance, the maximum minimum distance between two sets of points (Figure 5D–F). The Hausdorff distance between two trajectories ***X*** (composed of points ***x***) and ***Y*** (composed of points ***y***) is defined as

$$d_{H}\left(X,Y\right)=max\left\{\underset{x\in X}{\sup}\underset{y\in Y}{\inf}d\left(x,y\right),\underset{y\in Y}{\sup}\underset{x\in X}{\inf}d\left(x,y\right)\right\}$$

where *d* quantifies the Euclidean distance between ***x*** and ***y***, sup represents the supremum and inf the infimum.

To compare the Hausdorff distance between trajectories corresponding to different durations, we measured the Hausdorff distance between each trajectory and all other trajectories corresponding to the same direction and different durations (3 other duration bins) (Figure 5D).

To compare the Hausdorff distance between trajectories corresponding to different directions, we measured the distances between each trajectory and the trajectories corresponding to the 2 adjacent direction bins (one to the left and one right) that had the same duration. To test if the distances between trajectories corresponding to different durations were lower than trajectories corresponding to different directions, a *t*-test was performed for each recording (*N* = combinations of duration bins (3)× adjacent bins (2)× Durations (4) × Directions (8)=192).

To compare the Hausdorff distance between trajectories corresponding to different movements’ speeds(Figure 5F), we performed similar analyses. Because movement distance and speed were highly correlated (Figure 1–Figure Supplement 1), we selected movements that only were within a narrow window of distances (4–6 cm). We then divided these movements into high and low speeds and according to their direction (8 bins, 16 conditions total). We used the median speed in each dataset as a threshold to classify the movements. We scaled all trajectories to a reference duration and took the average for each condition. We projected the average neural activity of these 16 conditions into the subspace that encompassed direction and duration. To compare the distance between trajectories we followed the procedure described above for different durations and directions. Specifically, we compared the distance between high and low speed trajectories versus the distance between trajectories corresponding to adjacent direction bins (Figure 5F, left).

To test if distances between trajectories corresponding to different speeds were significantly lower than the distances between trajectories corresponding to different directions, a *t*-test was performed for each recording (*N* = Speed bins (2) × Adjacent bins (2) × Directions (8)=32).

### Decoding duration

To predict the trajectories’ duration from neural data, we built a linear decoder that used as input a reference neural trajectory that belongs to the same direction bin as the sample and whose duration is known. Since the reference and the sample trajectory are hypothesised to share their geometry, for each point in the sample *S*(*t*_*i*_) we can find the closest point in the reference trajectory

*R*(*idx*_*i*_), where the time index *idx*_*i*_ is calculated as:

(1) $$idx_{i}=\underset{t}{\arg\min}d\left(R\left(t\right),S\left(t_{i}\right)\right)$$

where *d* denotes the Euclidean distance. The relative speed (*v*) between the reference and sample trajectories was calculated as the slope of a linear regression between the times of the sample *t*_1*,…,n*_ and the *idx*_*i*_ values. Therefore, the duration of a sample trajectory can be predicted by the linear decoder: *Duration*_*sample*_ = *Duration*_*ref*_ *v*.

For all recordings, the 8 trajectories that corresponded to movements lasting between 300 and 400 ms were selected as the references. The remaining 24 trajectories were used as samples for which durations were predicted.

### RNN modelling

To examine how a population of neurons could control both the direction (the geometry) of a neural trajectory and its duration, we implemented a recurrent neural network in which its intrinsic dynamics are optimised to present rotational, stable outputs.

Since we observed the same key encoding features in PMd and M1, we implemented a single neural network that reproduced the separation and scaling of the trajectories. Further work could aim to reproduce the differences found between these two regions (such as the difference in timing in Figure 2) and analyse how they communicate.

We based our RNN on the stability-optimised circuit model of Hennequin et al. (2014), a rate-based network (*N* = 200 units, 100 excitatory, 100 inhibitory) whose temporal evolution is described by:

(2) $$\tau \frac{dx}{dt}=-x\left(t\right)+pI\left(t\right)+W\text{Δ}r\left(x,t\right)$$

where ***x*** is the vector of potentials, *τ* = 200 ms is the network’s time constant, *I* (*t*) is the ramping input signal, ***p*** is the vector defining the projection of the input to the units, ***W*** represents the connectivity matrix and ∆***r*** contains the instantaneous firing rate of all units with respect to a baseline.

We implemented the stability optimisation algorithm for ***W*** detailed in Hennequin et al. (2014), creating 20 realisations of ***W*** for which we obtained similar results to those shown for the example network in Figure 7.

The instantaneous firing rate ∆***r*** was defined as follows:

(3) $$\text{Δ}r\left(x\right)=\left\{\begin{array}{ll} r_{0}\tanh\left(x/r_{0}\right) & if\;x\leq 0\\ \left(r_{max}-r_{0}\right)\tanh\left(x/\left(r_{max}-r_{0}\right)\right) & if\;x>0 \end{array}\right.$$

with baseline firing rate *r*_0_= 5 Hz and maximum firing rate *r*_*max*_= 70 Hz.

For all of our simulations, following (Hennequin et al., 2014) we used a 1 s ramping exponential input *I* (*t*), whose time constant was fixed (200 ms), and a subsequent 200 ms decay (time constant= 0.1 ms). In our simulations, the start of the input (*t*= 1 s) is equivalent to the beginning of movement preparation, while the return to a steady-state corresponds to a time after movement ends. All simulations lasted 4 s. The strength of the input projection onto each unit was drawn from a uniform distribution between [−1, 1]. We also tested inputs drawn from a uniform distribution between [0, 1] to model solely excitatory inputs to the cortical circuit.

To simulate the effect of different directions, we selected one vector of input projection strengths ***p*** and rotated it 10 times to span 360° so that each input rotation corresponded to one direction bin. Each input, corresponding to a *n*-dimensional vector, was rotated in the high-dimensional space by independent rotations by the specified angle in each 2D plane taken sequentially from the *n*-dimensional vector.

We then defined the network’s output subspace by using PCA across the 10 resulting trajectories. We then measured the average distance between trajectories as described above. To vary the speed and duration of the trajectories we scaled the network’s time constant *τ*=[160, 200, 240] ms and projected the resulting trajectories onto the network’s output subspace. We defined the duration of each trajectory as the time it took for the trajectory to return to the region of recurrence, which was defined as above (radius *θ*=20, centred on the average start of the trajectories).

## Supplementary Material

Supplement 1 **Figure 1–Figure supplement 1.** Correlations between arm movement duration, speed, and distance
**Figure 2–Figure supplement 1.** Embedding dimensions for all recordings
**Figure 3–Figure supplement 1.** Period of recurrence for movements between 200–300 ms for all recordings
**Figure 3–Figure supplement 2.** Trajectories recur to the same region of the subspace for all movement durations
**Figure 7–Figure supplement 1.** Simulations for positive input projections
