# Supplementary material for: Motor cortex latent dynamics encode arm movement direction and urgency independently
Source: bioRxiv. 2023 May 26:2023.05.26.542452. Preprint. [Version 1] doi: 10.1101/2023.05.26.542452 (PMC10246015; doi:10.1101/2023.05.26.542452)
Supplement: Supplementary file 1 [file NIHPP2023.05.26.542452v1-supplement-1.pdf]

bioRxiv preprint doi: https://doi.org/10.1101/2023.05.26.542452; this version posted May 26, 2023. The copyright holder for this preprint (which was not certified by peer review) is the author/funder, who has granted bioRxiv a license to display the preprint in perpetuity. It is made available under aCC-BY 4.0 International license.

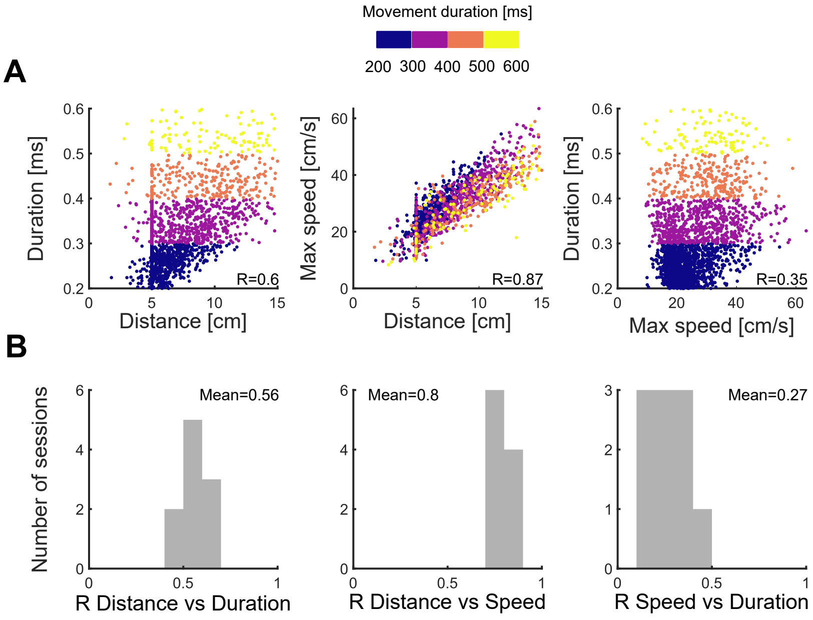

Figure 1–Figure supplement 1. A) Correlations between arm movement duration, speed, and distance for one example session. B) Histograms of correlation between arm movement duration, speed, and distance for all task sessions.

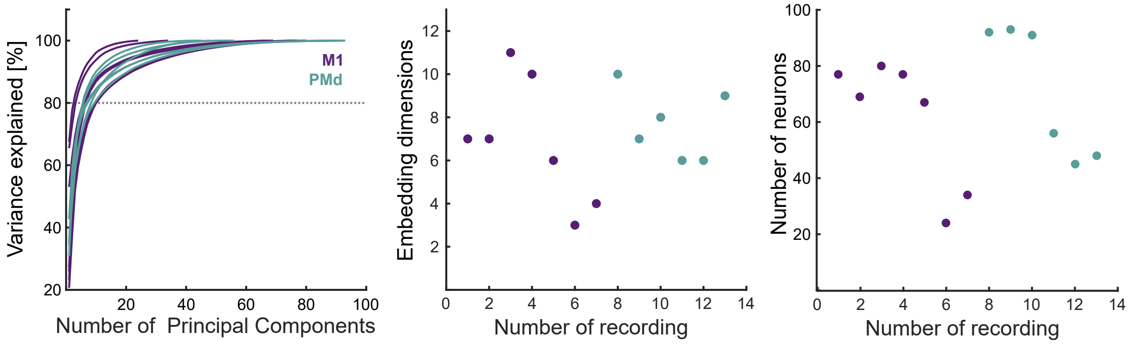

Figure 2–Figure supplement 1. Embedding dimensions for all recordings. Left: Variance explained as a function of the number of retained principal components. Centre: Embedding dimensions for 80% variance explained. Right: Number of neurons in each recording.

bioRxiv preprint doi: https://doi.org/10.1101/2023.05.26.542452; this version posted May 26, 2023. The copyright holder for this preprint (which was not certified by peer review) is the author/funder, who has granted bioRxiv a license to display the preprint in perpetuity. It is made available under aCC-BY 4.0 International license.

724

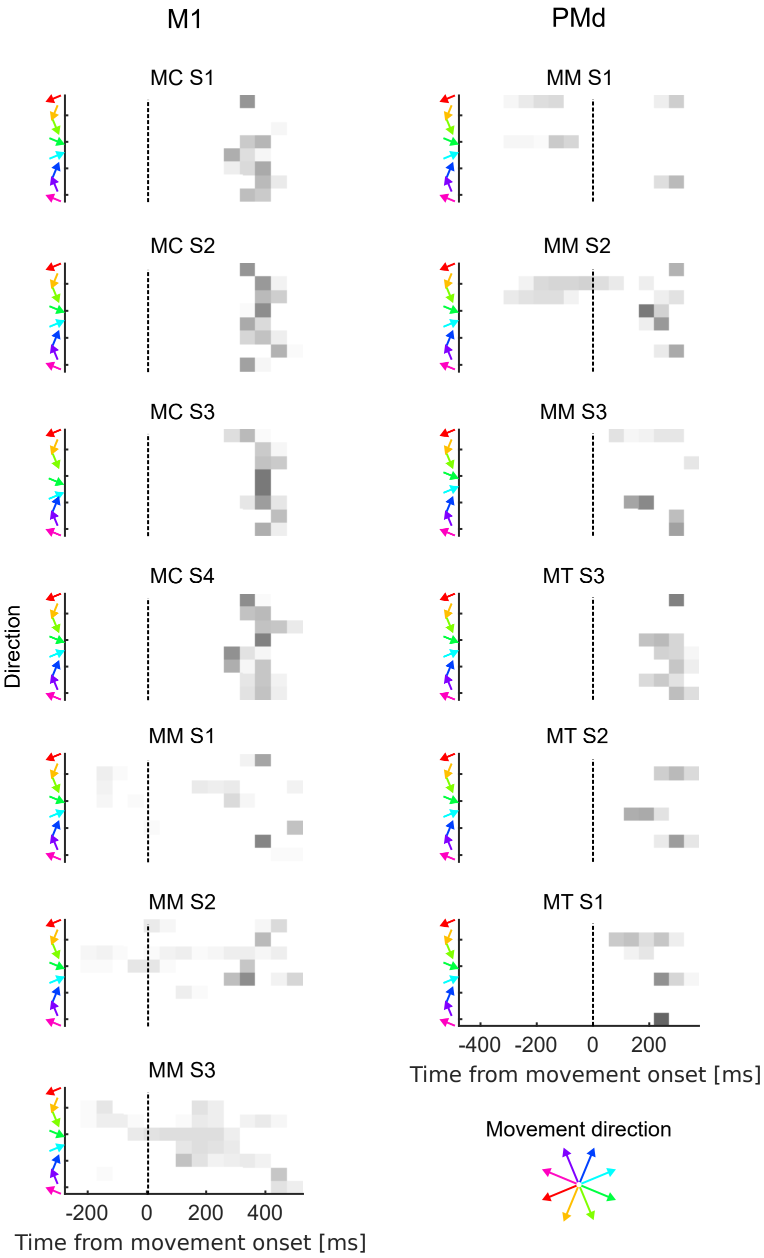

Figure 3–Figure supplement 1. Period of recurrence for movements between 200-300 ms for all recordings. Panel labels indicate monkey name (MX) and recording number (SN)

bioRxiv preprint doi: https://doi.org/10.1101/2023.05.26.542452; this version posted May 26, 2023. The copyright holder for this preprint (which was not certified by peer review) is the author/funder, who has granted bioRxiv a license to display the preprint in perpetuity. It is made available under aCC-BY 4.0 International license.

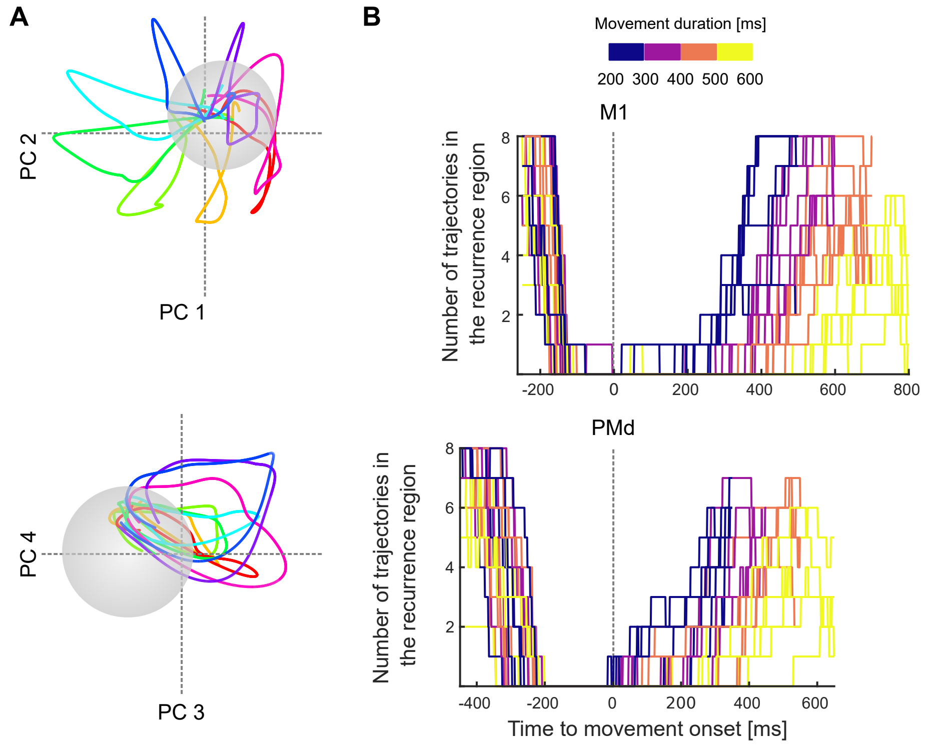

Figure 3–Figure supplement 2. Trajectories recur to the same region of the subspace for all movement durations. A) Schematic of recurrence region of subspace for one example recording. Only trajectories corresponding to one duration bin (200-300 ms) were selected to define the region of recurrence (grey sphere; see Methods). Lines are the average activity for each direction, for the duration bin used to define the region of recurrence. B) Number of trajectories inside the recurrence region at each moment in time, for M1 (top) and PMd recordings (bottom). Maximum is 8 (1 per direction bin).

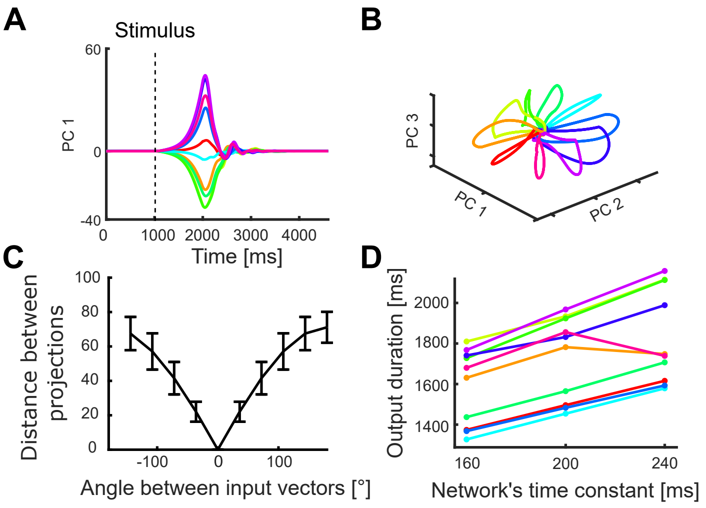

Figure 7–Figure supplement 1. Simulations for positive input projection $p \in [0, 1]$ A) Projection of RNN activity onto the first principal component (PC 1). B) Projection of RNN activity onto the first 3 PCs. C) Mean distance between trajectories by the angle between their input vectors. D) Output duration for different network time constants.
